# Prognostic model and optimal treatment for patients with stage IVc nasopharyngeal carcinoma at diagnosis

**DOI:** 10.1038/s41598-019-55586-w

**Published:** 2019-12-17

**Authors:** Yun-ming Tian, Wei-zeng Huang, Yu-hong Lan, Chong Zhao, Li Bai, Fei Han

**Affiliations:** 1 Department of Radiation Oncology, Hui Zhou Municipal Central Hospital, Guangdong Province, China; 2 Department of Medical Oncology, Hui Zhou Municipal Central Hospital, Guangdong Province, China; 3 Department of Radiation Oncology, Sun Yat-sen University Cancer Center; State Key Laboratory of Oncology in South China, Guangzhou, China

**Keywords:** Oncology, Radiotherapy

## Abstract

The treatment for patients with stage IVc nasopharyngeal carcinoma (NPC) at diagnosis was still controversial. In this study, we tried to build a prognostic score model and optimize the treatment for the patients. The prognostic model was based on the primary cohort involving 289 patients from 2002 to 2011 and the validation involving another 156 patients from 2012 to 2015.The prognostic model was built based on the hazard ratios of significant prognostic factors for overall survival (OS). By multivariate analysis, factors associated with poor OS were Karnofsky performance score ≤70, liver metastases, multiple-organ metastases, ≥2 metastatic lesions, lactate dehydrogenase >245 IU/I and poor response to chemotherapy (all P < 0.01). Based on these prognostic factors, patients were divided into the low-risk (0–2 points), intermediate-risk (3–6 points) and high-risk (≥7 points) groups. Five-year OS rates for the low-, intermediate- and high-risk groups were 49.3%, 9.7% and 0.0%, respectively (P < 0.01). Furthermore, loco-regional radiotherapy was associated with significantly better OS in low- and intermediate-risk patients, but not in high-risk patients. These results demonstrated that the prognostic score model based on six negative factors can effectively predict OS in patients with stage IVc NPC at diagnosis. Loco-regional radiotherapy may be beneficial for low- and intermediate-risk patients, but not for high-risk patients.

## Introduction

The epithelial malignancy nasopharyngeal carcinoma (NPC) is disproportionally common in Southern China^1^. Due to its proximity to an abundant lymphatic network, poorly differentiated and undifferentiated NPC have a higher propensity to metastasize than other squamous head and neck cancers^2,3^. The presence of distant metastasis is the most significant negative prognostic factor in NPC; median overall survival (OS) for patients with distant metastasis at diagnosis ranges from only 9 to 20 months^4–7^.

Patients with distant metastasis at diagnosis can have a different treatment response and prognosis compared to patients who develop distant metastasis after treatment^8–12^. However, the American Joint Committee on Cancer (AJCC) staging system classifies patients with distant metastasis as a single group, which does not enable stratification of prognosis^13,14^. Therefore, there is significant variation in the survival outcomes of patients who receive similar therapy. Moreover, the optimal treatment for patients with distant metastasis at diagnosis remains controversial. Systemic chemotherapy has been shown to be a critical component of comprehensive treatment and has a high objective response rate. However, the duration of disease control is limited when chemotherapy ceases^15,16^. The value of radiotherapy to the primary tumor is still uncertain, and the balance between survival benefits and radiation-related complications needs to be considered^17,18^. Therefore, it is necessary to create a risk-stratification system that enables the survival outcomes of patients with stage IV NPC at initial diagnosis to be predicted; this would have value from both a therapeutic and research point of view.

## Materials and Methods

### Patients

The inclusion criteria were: (1) patients with pathologically confirmed WHO type II or III NPC; (2) diagnosed with distant metastasis based on clinical symptoms, imaging finding and follow-up data; (3) who received at least one anti-cancer treatment including chemotherapy and/or radiotherapy. A total of 334 eligible cases were referred to Sun Yat-Sen University Cancer Center (278 cases) and Hui Zhou Municipal Central Hospital (56 cases) between January 2002 and June 2011; 45 patients were excluded due to missing clinical data (19 cases) or no treatment (26 cases). The remaining 289 patients were included in the primary cohort. One-hundred and fifty-six consecutive patients from 2012 to 2015 were included in the validation cohort.

Pre-treatment evaluation included a complete patient history, physical examination, complete blood count, renal and liver function tests, lactate hydrogenase (LDH), magnetic resonance imaging (MRI) of the nasopharynx and neck, chest radiographs or computed tomography (CT), abdominal sonography or CT, and single photon emission computed tomography whole-body bone scan. This protocol was in compliance with ethical standards and was approved by the institutional ethics committee of Sun Yat-sen University Cancer Center. Written informed consent was obtained from all participants. All methods were performed in accordance with the relevant guidelines and regulations.

### Treatment

The majorityof patients were treated with cisplatinum-based doublets: PF regimen (cisplatin + 5-fluorouracil), TP regimen (cisplatin + paclitaxel) and the TPF regimen (all three drugs). Due to poor medical condition or patient choice, some received mono-therapy.

Primary tumor radiotherapy was recommended for patients with distant metastasis in limited sites, stable metastatic disease after systemic chemotherapy, or serious symptoms caused by the primary tumor. Radiotherapy techniques ranged from conventional two-dimensional radiotherapy to three-dimensional conformal or intensity-modulated radiotherapy. Median dose prescribed to the primary tumor was 70.0 Gy. Local therapy for metastases included the palliative radiotherapy, radiofrequency ablation and surgery.

Treatment evaluations were performed after every two courses of chemotherapy, and then every 3 months after treatment. Tumor response was evaluated using the Response Evaluation Criteria in Solid Tumors (RECIST). Radiation toxicities were assessed and scored according to the radiation morbidity scoring criteria of the Radiation Therapy Oncology Group.

### Statistical analysis

OS was defined as the duration from date of diagnosis to death or censorship at last follow-up, calculated using the Kaplan-Meier method and compared using the log-rank test. Univariate and multivariate analysis were performed using the Cox proportion hazards model. Factors considered included patient factors (Karnofsky performance score, gender, age), basic laboratory characteristics (LDH, ALP, hemoglobin), and tumor features (T category, N category, features of metastases). A two-tailed P-value < 0.05 was considered significant.

The regression coefficients (β) of the independent prognostic factor were derived from the Cox regression equation (HR = e^β^), then converted into integers to provide a score index for the prognostic score model.

## Results

### Patient characteristics and survival

The characteristics of 289 patients in the primary cohort and 156 patients in the validation cohort are summarized in Table 1.Among the patients in the primary cohort,median age was 52-years-old (range, 15–75 years). The incidence of bone metastasis, liver metastasis and lung metastasis was 67.4% (195/289), 33.2% (96/289), and 15.9% (46/289), respectively. The rates of objective response (complete and partial responses), stabilization, and progressive disease were 56.1% (162/289), 32.2% (93/289), and 11.8% (34/289), respectively.

**Table 1:** Characteristics of the patients in the primary cohort and validation cohort.

Number of patients(%)		
Characteristic	Primary cohort (n = 289)	Validation cohort (n = 156)
**Gender**		
Male	249 (86.1)	129 (82.7)
Female	40 (13.9)	27 (17.3)
**Age (years)**		
Median	52	49
Range	15-73	24–77
**Karnofsky performance score (KPS)**		
>70	240 (83.0)	127 (81.4)
≤70	49 (17.0)	29 (18.6)
**T category**		
T1-2	100 (34.6)	20 (12.8)
T3-4	189 (65.4)	136 (87.2)
**N category**		
N0-1	81 (28.0)	22 (14.1)
N2-3	208 (72.0)	134 (85.9)
**Sites of metastasis**		
Bone	195 (67.4)	100 (76.3)
Liver	96 (33.2)	40 (25.6)
Lung	56 (17.6)	34 (21.8)
Others	20 (6.9)	18 (11.5)
**Multi-organ metastases (≥ 2)**		
Yes	71 (24.6)	50 (32.1)
No	218 (75.4)	106 (67.9)
**Numbers of metastatic lesions**		
1	56 (19.3)	31 (19.9)
2-5	105 (36.3)	42 (26. 9)
≥ 6	128 (44.4)	83 (53.2)
**Hemoglobin, Hb (g/L)**		
<120	39 (13.4)	17 (10.9)
≥120	250 (86.6)	139 (89.1)
**Alkaline phosphatase, ALP (IU/L)**		
≤110	224 (77.6)	126 (81.8)
>110	65 (22.4)	30(19.2)
**Lactate dehydrogenase, LDH (IU/L)**		
≤245	187 (64.7)	95 (60.9)
>245	102 (35.3)	61 (39.1)
**Chemotherapy cycles**		
1-3	86 (29.8)	39 (25.0)
≥4	203 (70.2)	117 (75.0)
**Chemotherapy response**		
CR+PR	162 (56.1)	88 (56.4)
SD	93 (32.2)	36 (23.1)
PD	34 (11.8)	32 (20.5)

Acute treatment-related toxicities were recorded. Grade III–IV leucopenia occurred in 47.4% (93/289) of patients during chemotherapy. Three patients died of treatment-related toxicities: one due to a severe infection caused by grade IV leucopenia, one due to gastric hemorrhage and another due to hepatic failure. Grade II-III mucositis occurred in 44.0% (80/183) of patients who received radiotherapy to the primary tumor.

Median survival time was 24 months (range, 2–144 months) and 25months (range, 4–86 months) for the primary cohort and validation cohort, and the 5-year overall survival rates were 23.0% and 24.3%,respectively.

### Univariate and multivariate analyses in primary cohort

The factors analyzed in univariate analysis are listed in Table 2. The negative prognostic factors for OS were a Karnofsky performance score (KPS) ≤ 70 (HR = 6.78, P < 0.01), N2-3 (HR = 1.30, P = 0.01), liver metastases (HR = 2.89, P < 0.01), multiple-organ metastases (HR = 2.76, P < 0.01), number of lesions (2–5: HR = 1.64, P = 0.04; ≥6: HR = 6.40, P < 0.01), lactate dehydrogenase (LDH) > 245 IU/I (HR = 3.11, P < 0.01), alkaline phosphatase (ALP) > 110 IU/I (HR = 2.15, P < 0.01) and poor response to chemotherapy (SD: HR = 1.87, P = 0.03; PD: HR = 7.12, P < 0.01).

**Table 2:** Univariate analysis of variables correlated with overall survival in primary cohort.

Factor	5-year OS (%)	HR (95% CI)	*P*-value
Gender, male/female	21.2/40.8	1.57 (0.94–2.64)	0.08
Age (years), ≤50/>50	22.5/25.8	1.43 (0.73–1.68)	0.76
KPS, >70/≤70	28.6/0.0	6.78 (4.17–9.25)	<0.01^a^
T category, T_1–2_/T_3–4_	24.9/22.3	1.13 (0.64–1.24)	0.41
N category, N_0–1_/N_2–3_	32.8/20.5	1.30 (1.09–1.56)	0.01
**Metastasis sites**			
Bone, no/yes	21.5/25.2	0.87 (0.61–2.04)	0.84
Liver, no/yes	32.4/8.5	2.89 (2.03–3.81)	<0.01^a^
Lung, no/yes	23.4/31.4	1.22 (0.82–3.81)	0.21
Others, no/yes	24.4/21.1	1.24 (0.70–2.20)	0.12
Multiple-organ metastases, no/yes	28.7/4.3	2.76 (1.98–3.84)	<0.01^a^
**Number of lesions**			
1	50.5	Baseline	—
2–5	35.7	1.64 (1.01–2.64)	0.04
≥6	3.5	6.40 (4.05–10.1)	<0.01
Hemoglobin, ≥120 vs. < 120 g/L	25.3/15.4	1.15 (0.78–1.70)	0.48
Serum LDH, ≤245 vs. >245IU/L	35.3/3.3	3.11 (2.24–4.20)	<0.01^a^
Serum ALP, ≤110 vs. >110IU/L	29.3/7.7	2.15 (1.59–2.93)	<0.01^a^
**Chemotherapy response**			
CR + PR	31.3	Baseline	—
SD	19.3	1.87 (1.25–2.32)	0.03^a^
PD	0.0	7.12 (4.68–10.8)	<0.01^a^

HR, hazard ratio; CI, confidence interval; ^a^ statistically significant.

Cox multivariate analysis identified a KPS ≤ 70 (HR = 6.78, P = 0.003), liver metastases (HR = 2.89, P < 0.01), multiple-organ metastases (HR = 2.76, P < 0.01), number of lesions (2–5: HR = 1.64, P = 0.04; ≥6: HR = 6.40, P < 0.01), LDH > 245 IU/I (HR = 1.69, P < 0.01) and poor response to chemotherapy (SD: HR = 1.87, P = 0.03; PD: HR = 7.12, P < 0.01) were negative independent prognostic factors for OS. N2–3 and ALP > 110 IU/I were not significantly associated with OS in Cox multivariate analysis (Table 3).

**Table 3:** Multivariate analysis of variables correlated with overall survival in the primary cohort.

Factor	HR (95% CI)	*P*-value
KPS, >70/≤70	3.48 (1.74–6.44)	<0.01
Liver metastases, no/yes	1.63 (1.32–2.53)	<0.01
Multiple-organ metastases, no/yes	1.52 (1.08–2.17)	<0.01
**Number of lesions**		
1	Baseline	—
2 to 5	2.31 (1.42–3.33)	0.03
≥6	3.33 (2.34–5.95)	<0.01
Serum LDH, ≤245 vs. >245 IU/L	1.69 (1.22–2.66)	<0.01
**Chemotherapy response**		
CR + PR	Baseline	—
SD	2.25 (1.42–3.09)	<0.01
PD	3.73 (2.31–6.98)	<0.01

### Establishment and validation of the prognostic model

**Figure 1:**
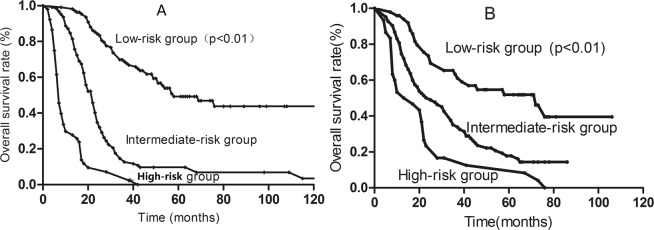
Kaplan-Meier survival curves for patients in the low-, intermediate- and high-risk groups in the primary cohort (**A**) and validation cohort (**B**).

The prognostic-score model was built based on the six negative independent prognostic variables. A score of 1, 2 or 3 was assigned for each factor according to the HR (n) value (Table 4). The maximum possible score for each patient was 12. The prognostic score for each patient was calculated by summing the individual scores for each factor. Patients were then assigned to three risk stratification groups: low-risk group (total score, 0–2; 38.5% of patients [111/289]); intermediate-risk group (total score, 3–6; 43.5% of patients [126/289]); and high-risk group (total score, 7–12; 18.0% of patients [52/289]). The median survival time of the low-, intermediate- and high-risk groups were 48.0, 22.0 and 7.0 months, respectively. The respective 5-year OS rates were 49.3%, 9.7% and 0.0%, respectively (P < 0.001; Fig. 1A) in the primary cohort. In validation cohort, the survival for three groups was also significantly different and the 5-year OS rates were 51.9%, 14.9% and 0.0%, respectively (P < 0.01; Fig. 1B).

**Table 4:** Prognostic index score based on patients in the primary cohort.

Characteristic	Score	*n* (HR = e^n^)
Multiple-organ metastases	1	0.42
Liver metastases	1	0.48
Serum LDH > 245 IU/L	1	0.52
**Number of lesions**		
1	Baseline	—
2 to 5	2	0.83
≥6	3	1.20
KPS ≤ 70	3	1.24
**Chemotherapy response**		
CR + PR	Baseline	—
SD	2	0.81
PD	3	1.30

### Response to treatment modalities in the three risk stratification groups

**Figure 2:**
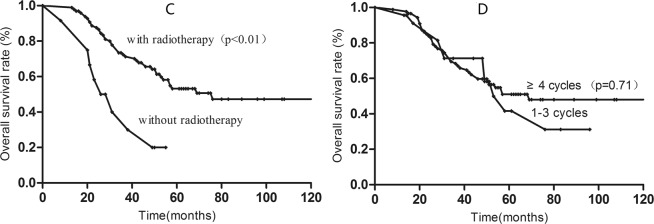
Kaplan-Meier survival curves for low-risk patients with radiotherapy (**C**) and chemotherapy cycles (**D**).

In the low-risk group, 5-year OS for patients treated with radiotherapy was 53.7%; no patients in this group survived more than five years without radiotherapy. However, patients who received 1–3 cycles and ≥4 cycles of chemotherapy achieved similar 5-year OS (41.7% vs. 51.0%; P = 0.71) (shown in Fig. 2).

**Figure 3:**
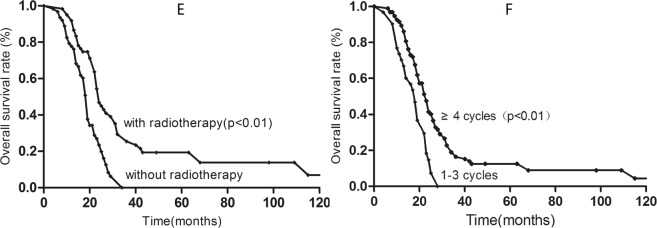
Kaplan-Meier survival curves for intermediate-risk patients with radiotherapy (**E**) and chemotherapy cycles (**F**).

In the intermediate-risk group, patients who received ≥4 cycles of chemotherapy achieved better OS than those who received 1–3 cycles (12.0% vs. 0%; P < 0.01). Furthermore, radiotherapy to the primary tumor was associated with better OS than no radiotherapy in the intermediate-risk group (19.4% vs. 0.0%; P < 0.01) (shown in Fig. 3).

**Figure 4:**
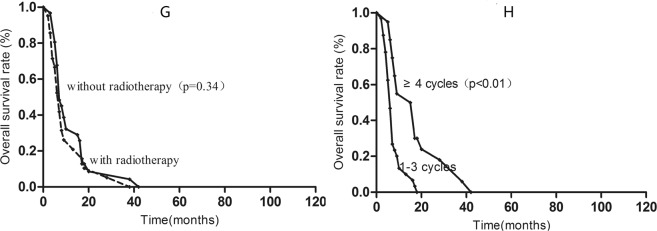
Kaplan-Meier survival curves for high-risk patients with radiotherapy (**G**) and chemotherapy cycles (**H**).

In the high-risk group, chemotherapy was an independent factor associated with OS: patients who received ≥4 cycles of chemotherapy had better 1-year OS than patients who received 1–3 cycles (13.4% vs. 50.0%; P < 0.01). However, radiotherapy to the primary tumor provided no benefit in terms of 1-year OS in the high-risk group (32.0% vs. 26.5%; P = 0.32) (shown in Fig. 4).

## Discussion

Traditionally, NPC with distant metastasis has a poor OS rate and is considered incurable. Yeh *et al*. reported the median OS duration of 125 patients with distant metastasis at diagnosis was only 9.7 months; 2-year OS was 14%^4^. However, the introduction of comprehensive treatment has greatly improved OS. Lin *et al*. retrospectively analyzed 105 patients treated with chemoradiotherapy: median OS duration was 25 months and estimated 5-year OS was 17%^5^. Furthermore, patients with distant metastasis at diagnosis have heterogenous treatment outcomes^17,18^. In a study of five patients who received comprehensive treatment, two had no evidence of disease at last follow-up (29 and 91 months) while the other three patients suffered disease progression within 12 months of the start of treatment^17^. Similarly, patients with different prognostic factors had variable survival outcomes in this study. The 5-year OS rates for patients in the low-, intermediate- and high-risk groups generated by the prognostic model were 49.3%, 9.7%, and 0.0%, respectively.

KPS is a significant prognostic factor in other tumor types, including lung cancer and esophageal cancer^19,20^. Poor performance status may be related to old age, severe comorbidity or more advanced disease. Jin *et al*. confirmed that poor performance status was associated with poorer survival in 718 patients with NPC with distant metastasis (hazard ratio of up to 2.1)^11^. Similarly, poor performance status was an important negative prognostic factor in this cohort of patients, with a hazard ratio of 3.48. Moreover, the 5-year OS rate for patients with a KPS >70 was 28.6%, while no patients with poor performance status survived for more than five years.

The sites and extension of distant metastases have long been considered to be prognostic factors in patients with NPC^11,18,21,22^. Compared to bone metastases and lung metastases, liver metastases are associated with poorer 5-year OS (32.4% vs. 8.5%). Ong *et al*. proposed a prognostic scoring system for OS after retrospectively reviewing 220 patients with distant metastasis, though only 21% of these patients had metastases at initial diagnosis. Liver metastasis was negatively associated with OS (hazard ratio, 1.54) in their prognostic model^11^. Consistent with these previous studies, we found patients with multiple-organ metastases or more than one lesion had poorer OS than those with single-site or single metastases. The 5-year OS rates for patients with 1, 2–5, and ≥6 lesions were 50.5%, 35.7%, and 3.5% respectively. These observations could be explained by the theory of oligometastases, a status extending from disease that remains localized to wide-spread dissemination that can be potentially cured^23–25^.

Our prognostic model based on the six independent negative factors may help to predict survival outcomes and identify patients who may benefit from comprehensive treatment. Systemic chemotherapy remains the recommended first-line treatment for NPC and is the most commonly used strategy in patients with distant metastasis at diagnosis. The value of loco-regional radiotherapy to the primary tumor remains a major controversy. However, increasing evidence indicates loco-regional radiotherapy provides a survival benefit by reducing primary tumor bulk and the severity of symptoms caused by the tumor^4–7,17,18^. Hu *et al*. retrospectively 41 patients treated with systemic chemotherapy followed by loco-regional definitive intensity-modulated radiation therapy as a first-line treatment; the 5-year OS rate was 22.5% and five patients were alive without evidence of disease after 52 to >101 months^18^. Partly consistent with those results, we found loco-regional radiotherapy could significantly prolong the survival of patients in the low- and intermediate-risk groups, but not the high-risk group. Furthermore, the survival benefits of systemic chemotherapy were variable. Patients in the intermediate- and high-risk groups who received at least four cycles of chemotherapy achieved significantly better OS than those who received less than four cycles, while the number of cycles of chemotherapy had no significant effect on OS in low-risk patients.

There were several weaknesses in the study. First, this prognostic model was based on retrospective analysis with inevitable selection bias in the treatment modalities. Second, for the lack of serum EBV DNA data, we did not investigate it in the study which has been demonstrated as an independent prognostic factor in disseminated NPC^25^. Finally, the data used to build the model was from endemic area which was need to be validated in the non-endemic area.

In conclusion, our prognostic score model based on six negative independent prognostic factors can effectively predict the survival of patients with NPC who have distant metastasis at initial diagnosis. Furthermore, loco-regional radiotherapy may be necessary for low-and intermediate-risk patients, but not for high-risk patients. However, due to the selection from the retrospective analysis, more prospective studies are needed to validate the prognostic score model.
